# Spatial and time domain analysis of eye-tracking data during screening of brain magnetic resonance images

**DOI:** 10.1371/journal.pone.0260717

**Published:** 2021-12-02

**Authors:** Abdulla Al Suman, Carlo Russo, Ann Carrigan, Patrick Nalepka, Benoit Liquet-Weiland, Robert Ahadizad Newport, Poonam Kumari, Antonio Di Ieva

**Affiliations:** 1 Computational NeuroSurgery (CNS) Lab, Faculty of Medicine, Health, and Human Sciences, Macquarie University, Sydney, Australia; 2 School of Psychological Sciences, Faculty of Medicine, Health, and Human Sciences, Macquarie University, Sydney, Australia; 3 Centre for Elite Performance, Expertise and Training, Macquarie University, Sydney, Australia; 4 Department of Mathematics and Statistics, Faculty of Science and Engineering, Macquarie University, Sydney, Australia; UMR8194, FRANCE

## Abstract

**Introduction:**

Eye-tracking research has been widely used in radiology applications. Prior studies exclusively analysed either temporal or spatial eye-tracking features, both of which alone do not completely characterise the spatiotemporal dynamics of radiologists’ gaze features.

**Purpose:**

Our research aims to quantify human visual search dynamics in both domains during brain stimuli screening to explore the relationship between reader characteristics and stimuli complexity. The methodology can be used to discover strategies to aid trainee radiologists in identifying pathology, and to select regions of interest for machine vision applications.

**Method:**

The study was performed using eye-tracking data 5 seconds in duration from 57 readers (15 Brain-experts, 11 Other-experts, 5 Registrars and 26 Naïves) for 40 neuroradiological images as stimuli (i.e., 20 normal and 20 pathological brain MRIs). The visual scanning patterns were analysed by calculating the fractal dimension (FD) and Hurst exponent (HE) using re-scaled range (R/S) and detrended fluctuation analysis (DFA) methods. The FD was used to measure the spatial geometrical complexity of the gaze patterns, and the HE analysis was used to measure participants’ focusing skill. The focusing skill is referred to persistence/anti-persistence of the participants’ gaze on the stimulus over time. Pathological and normal stimuli were analysed separately both at the “First Second” and full “Five Seconds” viewing duration.

**Results:**

All experts were more focused and a had higher visual search complexity compared to Registrars and Naïves. This was seen in both the pathological and normal stimuli in the first and five second analyses. The Brain-experts subgroup was shown to achieve better focusing skill than Other-experts due to their domain specific expertise. Indeed, the FDs found when viewing pathological stimuli were higher than those in normal ones. Viewing normal stimuli resulted in an increase of FD found in five second data, unlike pathological stimuli, which did not change. In contrast to the FDs, the scanpath HEs of pathological and normal stimuli were similar. However, participants’ gaze was more focused for “Five Seconds” than “First Second” data.

**Conclusions:**

The HE analysis of the scanpaths belonging to all experts showed that they have greater focus than Registrars and Naïves. This may be related to their higher visual search complexity than non-experts due to their training and expertise.

## Introduction

Radiological imaging is the diagnostic workhorse in medicine. Radiologists screen medical images to detect pathologies. However, diagnosis can be susceptible to human errors causing missed recognition of pathologies [1–3]. Therefore, there has been considerable research focused on medical diagnostic errors in recent years [4–7]. A large amount of research related to radiologists’ eye movements has been carried out using eye-tracking data to find the relationship between expert radiologists’ search patterns and diagnostic expertise (e.g., recognition of abnormal findings in the radiological images) [8–12]. Kundel *et al*. examined the eye-gaze patterns of radiologists when searching for lung cancers in chest radiographs and identified three types of errors that occur when searching a medical image for pathology: (1) visual search errors, where they never fixate on the abnormality (30%); (2) recognition errors, where the abnormality is fixated but may be camouflaged in the image and therefore no meaning is assigned to the area (25%); and (3) decision errors, where the abnormality is fixated but actively dismissed as an abnormality (45%) [13].

Most studies related to eye-tracking were performed by comparing the gaze behavior of experts and novices [10, 14–18]. Krupinski [16] examined the radiologists’ experience on gaze behaviour by comparing scanpaths between three expert mammographers and three radiology residents. The author investigated dwell time between the two groups and found that non-experts spent more time on image searching with larger spatial coverage. Kundel *et al*. [18] proposed the concept of global perceptual processing during radiologic image reading. They reported that expert radiologists develop global processes for image reading, which guides them to find abnormalities more efficiently than less experienced counterparts. Wood *et al*. [17] performed an eye tracking study on radiologists with expertise in skeletal imaging who varied in level of experience. They found that consultant radiologists demonstrated superior pattern recognition skills compared with naïves and trainees. Kelly *et al*. [10] studied the progression of radiologic interpretation skill in chest radiographs by comparing diagnostic accuracy and visual scanpaths among four clinical groups. The difference between groups was greater for diagnostic accuracy compared with visual search metrics. Alamudun *et al*. [15] investigated the relationship between image readers’ characteristics and the images by analysing gaze complexity of readers in mammographic mass detection. They used fractal analysis to quantify visual search and receiver operator characteristic (ROC) for diagnostic decisions analysis to differentiate the participant groups and found that gaze complexity is dependent on the image reader characteristics and properties of images.

The bulk of the research on radiologists’ visual scan patterns has focused on breast [19–21] and chest image interpretation [12]. However, there is significantly limited eye-tracking research related to the interpretation of brain images [14, 22, 23]. Cavaro *et al*. [22] investigated the glioma diagnosis process using multiple MRI sequences by analysing temporal fixation organisation, number of returns to a location, and saccade length. They found clear steps which radiologists normally follow sequentially and systematically during diagnostic image scanning. Matsumoto *et al*. [23] investigated neurologists’ visual attention during CT screening of brain cerebrovascular diseases. They compared gaze distribution (by means of heat maps) with image saliency maps between neurologists and controls. They also compared dwell time in regions of interest (ROI). They found that neurologists used “top-down saliency” more effectively than control subjects though both groups used “bottom-up salience”. In addition, they reported that the dwell time was similar for both groups on large lesions but neurologists spent more time than controls on clinically important areas with low salience. Crowe *et al*. [14] analysed eye movements in different clinical groups using ScanMatch gaze comparison techniques in a brain tumour detection task for both 2D and 3D cases. In their findings, experts showed highest scanning pattern similarity in both cases. In the 2D case, gaze patterns were most similar within experts for correct diagnosis, and the patterns were least similar for false negatives for all groups.

Traditional research is well established using radiologist visual scanning patterns on non-brain stimuli [12]. Most studies investigated the visual characteristics such as total dwell time, time to initially hit true lesions, number of hits, overall search times, dwell time for abnormal regions and spatial complexity of gaze [15]. In addition to the parameters studied in those works, we propose a computational approach applied to spatiotemporal dynamics which we deem to have a critical role in radiology. However, there are no known studies which considered temporal and spatial features simultaneously. Given that spatial shape complexity and time series analysis are simultaneously required to completely characterise the gaze of radiologists, the previously quoted work may not have depicted a complete profile of radiologist gaze behaviour. Moreover, the limited research investigating eye-tracking with brain stimuli only analysed scanpath comparisons through a similarity score, number of returns to a location, temporal fixation organisation, saccade length and heat maps [14, 22, 23]. Importantly, the spatial shape complexity of radiologists’ gaze in breast or chest images is different from brain images due to the different anatomical and pathological spatial structure of the brain. Therefore, the analyses for breast or chest are not directly translatable to brain imaging cases.

To overcome such limitations, the current study aims to quantify the visual search characteristics of radiologists by taking spatial shape complexity and time series analysis into account simultaneously. In our experiments, we aim to characterise gaze patterns of radiologists with other medical experts in brain science. We use FD to measure scanpath spatial geometrical complexity, and the HE to quantify the persistency/anti-persistency of a participant’s gaze on the stimulus over time. The HE analysis of time series can only provide a rough estimate of the scanpath focusing status, proving inadequate for detailing movement complexity. In particular, it can only reveal the focusing ability in terms of spatial regions but not details about how the gaze moves around them. For this reason, the spatial and temporal analyses are simultaneously required to characterise gaze behaviour properly.

In our study, radiologist attention is seen to gravitate more towards pathological stimuli. Specifically, Brain-experts (e.g., neuroradiologists and neurosurgeons) demonstrate more expertise than Other-experts in radiology due to their more domain specific knowledge. This matched our expectation that a suspicion of pathology will lead to greater scrutiny during diagnosis. An opposite pattern emerges as attention increases when searching for abnormalities with low salience over time in normal stimuli.

The image interpretation process in a clinical setting is typically performed with stacks of 2D and volumetric MR images. However, we specifically used 2D images from different projections (i.e., axial, coronal and sagittal) to perform the analyses because both dynamic stacks and static 2D image viewing are used for medical image perception tasks [14], and for training as well. Moreover, radiologists usually spend more time on a few 2D images rather than other images of the stack. Our pathological stimuli were selected from the specific type of images which radiologists use for diagnosis. Visual tracking while viewing the dynamic stack will not only create experiment complexity but may also reduce confidence within experimental results. Therefore, the visual tracking on a single snapshot can be considered a good surrogate to characterise radiologist gaze patterns.

## Materials and methods

### Participants

#### Expert participants

Twenty-seven radiologists and radiology registrars were recruited from a large radiology conference hosted in the United States (Radiological Society of North America). Five additional neurosurgeons and neurosurgery registrars were recruited from an Australian University. One participant was removed from analysis because their eye-tracking ratio was below 30% for the session, resulting in a total sample of 31 participants with expertise in medical imaging (9 Female, *M* = 44.58 years, *range* = 29-75 years). The expertise of the radiologists ranged from Registrars (resident/trainee) (*N* = 5), Other-experts (*N* = 11), and Brain-experts (*N* = 15). For the purpose of our study, all neurosurgeons and neurosurgery registrars were classified as Brain-experts. Radiology expertise was determined by whether the participant held a self-reported board certification or other post-graduate radiology qualification. See Table 1 for a descriptive summary of the expert sample. The Other-experts reader type was designated for the non-brain imaging experts.

**Table 1:** Participants’ characteristics summary.

Reader type	Experience level	No. of participants
Brain-experts	≥ 10 years of practice	15
Registrars	≤ 2 years of training	5
Naïves	N/A	26
Other-experts	≥ 10 years of practice	11

#### Naïve participants

Twenty-six individuals were recruited from the Macquarie University undergraduate community (18 Female, *M* = 22.36 years, *range* = 18-33 years). Participants were recruited either via word-of-mouth or through the Department of Psychology’s recruitment portal. Participants who signed up through the portal received course credit as compensation for completing the study.

All participants reported normal or corrected-to-normal vision, had no prior experience reading medical images and were naïve to the purposes of the experiment.

### Stimuli

**Fig 1:**
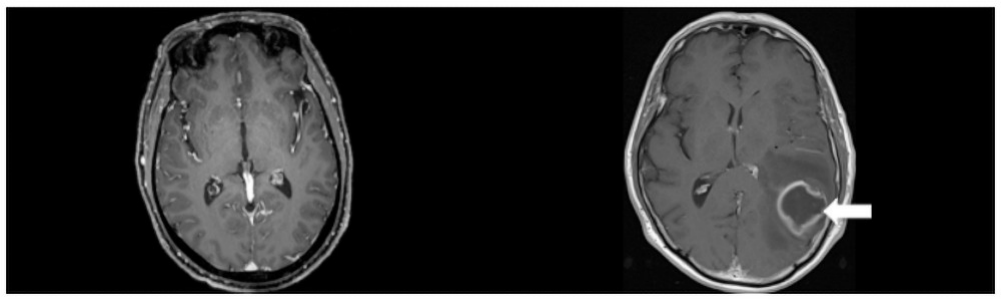
Exemplary stimuli used in our study, showing T1-weighted post-contrast MR images of a normal (left image) and a pathologic (right image) brain. The arrow indicates a glioblastoma. Note, the arrow was not shown to participants.

The stimuli consisted of a set of 40 de-identified brain magnetic resonance images (MRIs: 50% normal). Different projections (i.e., axial, coronal and sagittal) as well as different sequences (e.g., T1, T1 post-contrast, etc.) were included in the stimuli dataset. Fig 1 shows two sample images.

### Apparatus

Stimuli were presented on a 15.6’ laptop with a 1920 × 1080 display resolution operating at 60 Hz (Dell Precision M4800, Dell Inc., USA). Ocular movements were measured using a SMI RED250mobile Eye Tracker (SensoMotoric Instruments, Germany), sampled at 250 Hz. For each participant, the eye tracker was calibrated using a 9-point calibration procedure. During calibration and throughout the experiment, participants kept their head rested on a chin rest positioned 60 cm from the laptop display. The laptop display subtended a maximum visual angle of 32°.

### Procedure

**Fig 2:**
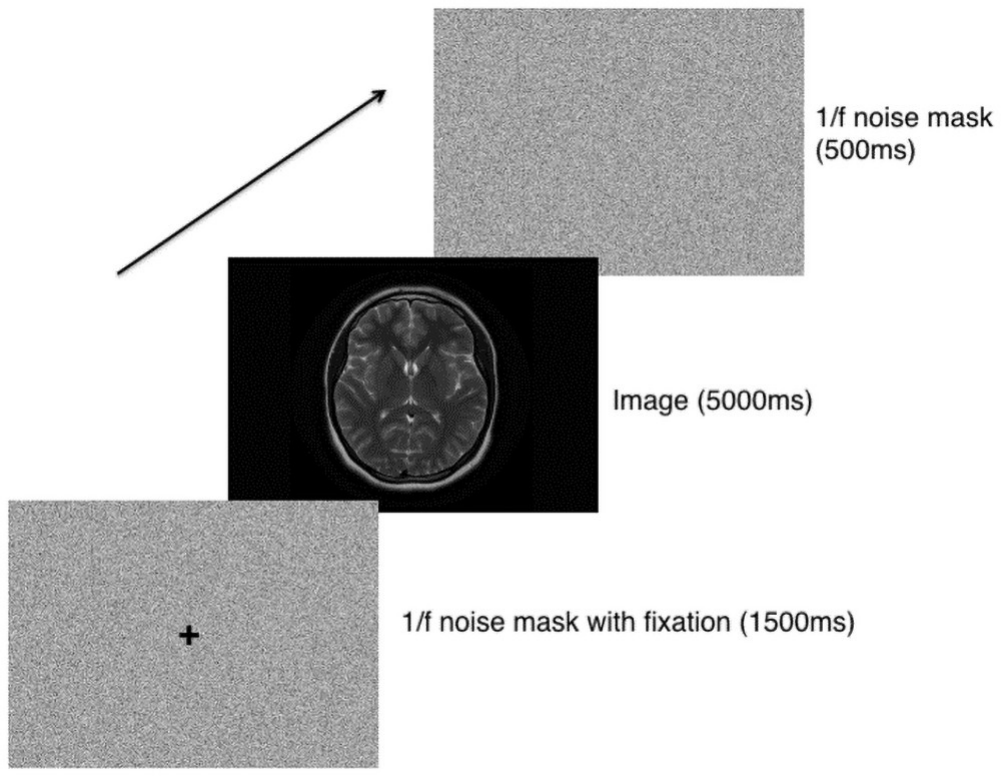
Data collection procedure.

Following informed consent, participants completed demographic questions regarding age, radiological expertise in reading brain MRIs (if any), and the number of radiological cases reviewed in an average day and over the past 12 months. Afterwards, participants were asked to keep their head still using a chin rest. Participants were informed that they would view a series of images and were told to look at the images. The exception to this was when a fixation cross was presented, in which case participants were told to keep their gaze fixated centrally on the cross (see Fig 2). There was no task for participants other than viewing the images. Each sequence of images consisted of a 1/f noise mask with cross fixation (1500 ms), a target image (5000 ms), and then a 1/f noise mask (500 ms). The order of target stimuli that were presented was randomised and the same order was used for all participants. The right eye’s data of the participants were used in this study.

Participants were recruited as part of a larger study investigating oculomotor behaviours when viewing visual stimuli from different categories. Thus, the sequence of brain MRI stimuli was presented as the second block as part of this larger study. This sequence was preceded by a block consisting images relating to paintings and natural scenes which followed the same trial presentation order as shown in Fig 2. Each procedure took 15 minutes to complete. The study design and procedure were approved by the Macquarie University Institutional Review Board.

### Data processing

The scanpaths were obtained by connecting time-ordered saccades and fixations, resulting in dense spatial patterns. The FD of spatial analysis, and R/S and DFA of the time series analysis were calculated from the scanpaths.

#### Fractal dimension (FD)

The scanpath can be considered as a natural complex object that does not follow any regular geometric shapes. Prior research suggests that expert radiologists develop a global perceptual processing capability and produce scanpaths that are systematic and sequential during diagnostic image scanning [18, 22]. Therefore, expert radiologists create approximately similar spatial shapes and temporal orders in their scanpaths. On the other hand, naïves’ scanpaths may lack defined temporal and spatial properties due to their lack of expertise in radiology. To measure scanpaths quantitatively, Euclidean geometry is not appropriate due to the irregular geometric shapes of the scanpaths. Although scanpath length may be calculated, it is not suitable for differentiating expert from non-expert gaze. This is because the length of an expert scanpath may be similar to the length of a non-expert’s scanpath, but be different in shape. Therefore, the shape of the gaze is important for characterising radiologists compared to naïves. In spite of the fact that the scanpath is not a mathematical nor a natural fractal, fractal analysis is a viable option to quantify it, as it is an irregular object [24]. Fractal geometry is the branch of mathematics that describes ‘rough’, infinitely complex, and branching objects [25]. It can be seen as a universal language for describing nature quantitatively, with several applications found in medicine and in the neurosciences [26–28]. The geometrical complexity of the patterns can be measured by the fractal dimension (FD), which is a non-integer scalar parameter quantifying the geometrical complexity of objects and/or patterns [24]. Any small changes in FD can correspond to big differences in the shapes due to involvement of logarithmic calculations. The FD values are in the range from 0 to *D*_*E*_, where *D*_*E*_ is the Euclidean dimension of the space containing the fractal shape or pattern. However, the range is (*D*_*E*_ − 1 < *FD* ≤ *D*_*E*_) for continuous fractal patterns, as the scanpath is a 2-dimensional representation of a path visualised on a plane, therefore *D*_*E*_ = 2. Lower FD values corresponds to lower occupation of the space in which the object is embedded, with higher values signifying higher space-filling occupancy. There are many methods for computing FD, of which, the most used ones are the box-counting and compass methods [29–31]. In this work, we have used the compass method as it works better with time series data and can measure geometrical complexity in a small spatial coverage which is important in this application since radiologists gaze may be confined within a small spatial area. We applied the compass method after converting the original X-Y cartesian points (original gaze) to a 1D plot of X versus Y (transformed gaze (TG)). The FD is defined mathematically as follows:

$$\begin{array}{c} \begin{array}{c} FD_{compass}\left(TG\right)=\underset{\alpha \to 0}{\text{lim}}\frac{\text{log}N(\alpha )}{\text{log}(1/\alpha )} \end{array} \end{array}$$ (1)

where *N*(*α*) is number of circles of length *α* to cover the TG scanpath.

**Fig 3:**
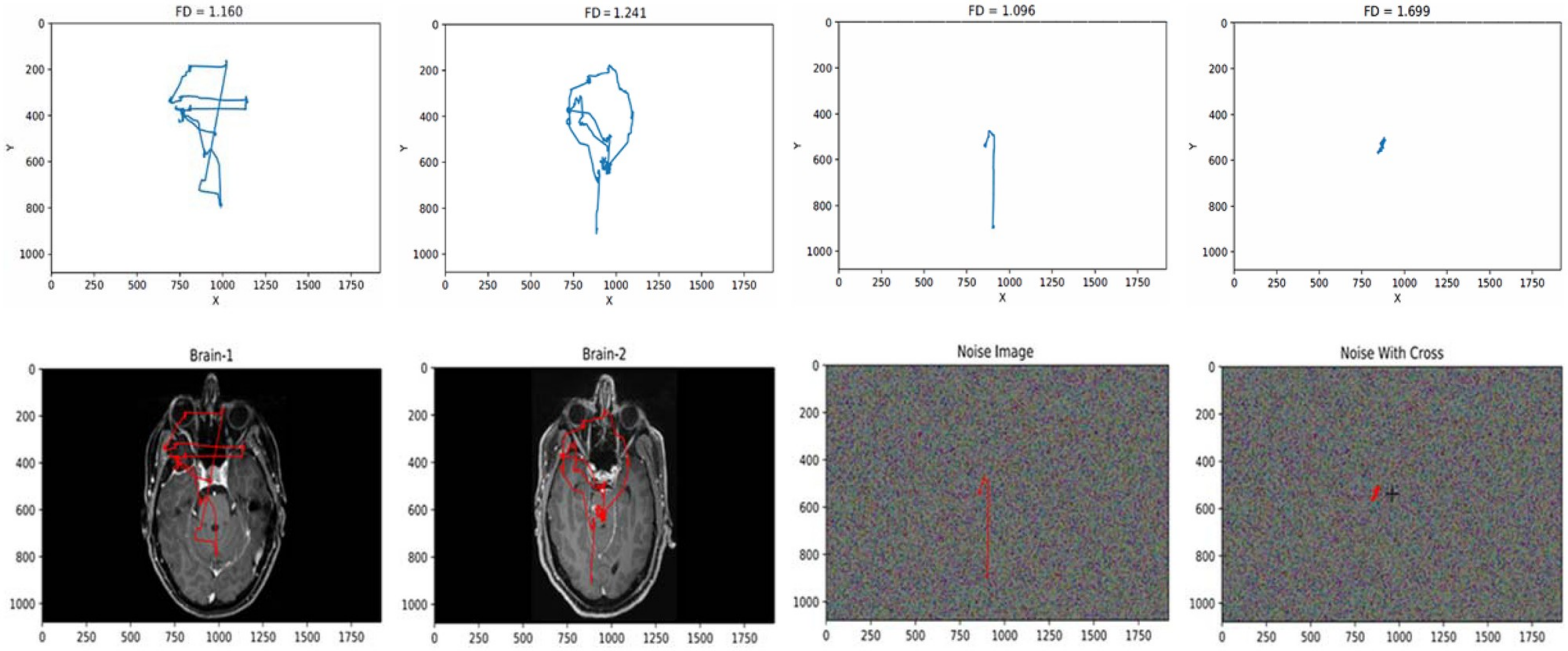
Some sample scanpaths with fractal dimension (FD) values and corresponding stimuli explaining the relation between FD values and geometric complexity.

Fig 3 shows some scanpaths with FD values and stimuli. The path for “Noise With Cross” stimulus has high FD values but has low space occupancy, which is due to the fact that the scanpath’s points are located within a very small space with small movements owing to clear target resulting high geometrical complexity.

#### Time series analysis

**Fig 4:**
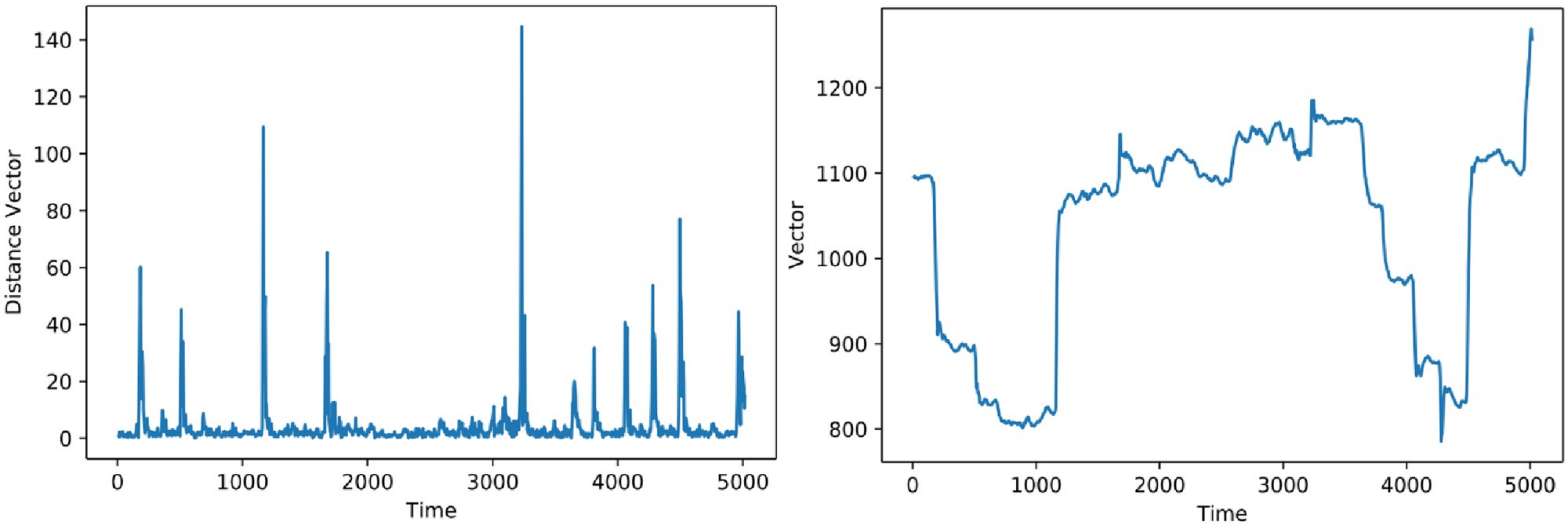
Time series illustrating distance vector’s suitability for time series analysis compared to vector. The vector versus time plot has constant amplitude effect and does not show participant’s perfect gaze behaviour in relation to the region of interest (ROI).

Time series analysis of the scanpath is also of paramount importance for characterising expertise as experts try to find abnormality and focus on it, whereas registrars scan patterns are less correlated over time, as shown in our experiment (see below). This analysis can measure the focusing ability of a participant during the task of viewing a stimulus. In this study, the time series was represented as a distance vector versus time. The distance vector was selected as it can help to measure long-term memory of a participant without any artefacts. On the other hand, if a vector is used in the time series, it will create a constant amplitude in the time series, as participants always start their visual search from the center of the stimulus as discussed in the data collection protocol. In addition, the constant amplitude effect remains in the series as participants rarely search in the origin region of the stimulus (see Fig 4). The distance vector was measured as the distances between two consecutive points of the scanpath, while the vector was the distance from origin to the scanpath point, regardless of the previous points. Moreover, the distance vector provides exact eye movement variations in the time series. But the vector cannot do these variations along the arc. This enables the evaluation of the persistence/anti-persistence of the participant gaze on the image points over time.

Although conventional parameters provide valuable radiological information, for example, time to initially hit true lesions, number of hits, dwell time for abnormal regions, these features cannot characterise completely radiologists’ behaviour in terms of global perception because those parameters do not consider full length of scanpaths. The scanpath for total time has to be considered to characterise the global perception. In this study, we have analysed the time series for the total period using the Hurst Exponent (HE).

The HE can provide the long-term memory of a time series and it relates to auto-correlations of the series. This analysis can measure the strength of participants’ memory for past events [32, 33]. Its values range between 0 and 1, and are proportional to the focusing status of a scanpath. The range 0.5 < *HE* ≤ 1 means that the series will have less fluctuations, approaching to 1 resulting smooth appearance of the series and converging participants’ gaze to a small spatial region (i.e., more focusing). The range 0 ≤ *HE* < 0.5 indicates that the series will have high fluctuations, close to 0 resulting in a rough appearance of the series. The *HE* = 0.5 means that the series will be completely random or Gaussian Brownian motion, thus suggesting that there will be no specific area attracting the participant gaze. There are many methods for estimating the exponent using both in the time domain and frequency domain such as R/S, DFA, periodogram regression, local Whittle’s estimator, aggregated variances, wavelet analysis. In this study, the R/S and DFA methods were used to analyse the time series. The R/S is the oldest and best-known method. The HE is defined in terms of asymptotic behaviour of R/S method as follows:

$$\begin{array}{c} \begin{array}{cc} E[\frac{R(n)}{S(n)}]=Cn^{HE} & \;\text{as}\;n\to \infty \end{array} \end{array}$$ (2)

where.; E[-] represents expected value, *R*(*n*) is the range of first *n* cumulative deviations from mean, *S*(*n*) is their standard deviation, *C* is a constant, *n* is the number of observed data points in the time series.

However, prior research suggests that the scanpaths have long-range power-law positive correlations [34]. As a result, the scanpath time series may contain a steady trend or multiple trends over a large time scale of observation and become non-stationary. In this case, a bias is introduced in the R/S exponent [35]. To avoid such bias, DFA exponent is used to analyse the time series. In the DFA method, the exponent is calculated by removing the large-scale trend with calculating fluctuation within lower scale of observation. The R/S exponent is a kind of DFA for which the trend is constant. The values of DFA exponent have similar meaning like HE for the range of 0 ≤ *HE* ≤ 1 except that the series is non-stationary for *DFA* > 1 and has *HE* = *DFA* − 1.

### Statistical analysis

We used a linear mixed model with a random intercept among the different participant groups to examine whether there is a significant difference for all the visual characteristics as our experiments contain hierarchical data with repeated measures for each participant. The dependent variable was examined at the stimulus level. In particular, the FD, R/S and DFA of each subject for each stimulus were used as dependent variables in our statistical analysis. The linear mixed model also treats our unbalanced sample sizes of the participants’ groups as mixed model does not require a balanced design. A planned comparisons design was employed for our statistical analysis. We grouped the 57 participants into five groups to analyse the visual characteristics: Brain-experts, Registrars, Naïves, Other-experts and All-radiologists. The All-radiologists consists of Brain-experts, Registrars and Other-experts. As our main purpose was to see the difference between experts and non-experts groups, we performed the statistical test for the five pairs of Brain-experts-Registrars, Brain-experts-Naïves, Other-experts-Registrars, Other-experts-Naïves, and All-radiologists-Naïves. The *p* values are reported after adjustment using false discovery rate (FDR) method by considering the five tests. Effect size for each statistical test was measured using Cohen’s D method. Our statistical analysis was performed by using the statistical program R, version 4.1.1.

## Results

**Fig 5:**
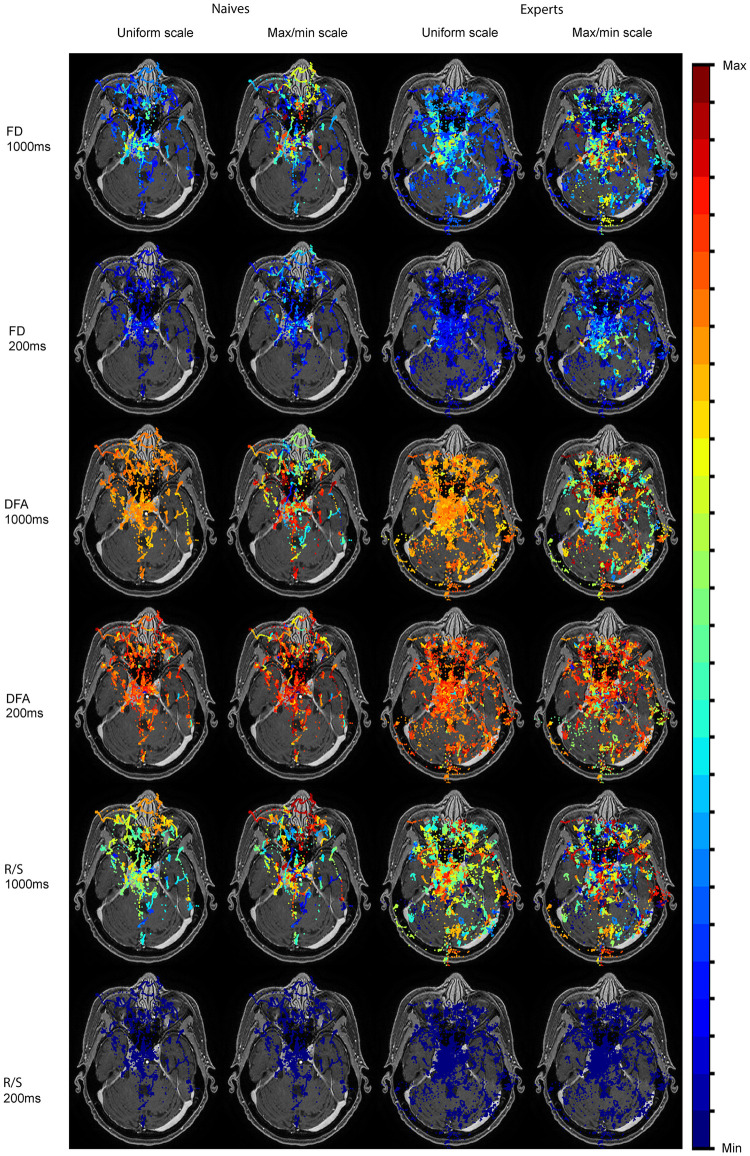
Heat maps of FD, R/S and DFA for a stimulus using eye-tracking data from Naïves and expert (Brain-experts and Other-experts) groups. The parameters were evaluated by sliding with time slots of 1000 ms and 200 ms over the full viewing duration of 5000 ms. The values of the parameters were uniformed for the uniform scale while max/min scale keeps the original values for each participant.

We did an initial analysis by generating heat maps of the visual search parameters (FD, R/S, and DFA) for each stimulus using data from each participant and group of participants to know where participants were looking. Fig 5 shows heat maps for a sample stimulus using tracking data from Naïves and expert groups. As shown in Fig 5, experts were viewing brain areas, spending a longer time looking for anomalies there, while Naïves’ gaze falls on many non-relevant areas (e.g., nose, eyes, etc.). Subsets of 200 ms and 1000 ms time slots taken by sliding 100 ms from the full scanpath have been evaluated here to generate the heatmaps. The parameters evaluated using the small time slot have not enough data to evaluate the parameters properly. Therefore, the heat maps using 200 ms seemed to be noisy.

We did two types of analysis of the visual parameters: “First Second” and “Five Seconds” as most abnormalities are detected in the “First Second” of viewing [3, 36]. Also, these types of analysis were used in other fields (e.g., neuroscience of aesthetics [37]). For the “First Second” analysis, we considered the initial gaze points related to first second duration. The full scanpath of 5 seconds duration was used for the “Five Seconds” analysis. The results were analysed for the pathological and normal stimuli separately.

### Fractal dimension

#### Analysis for “First Second”

**Fig 6:**
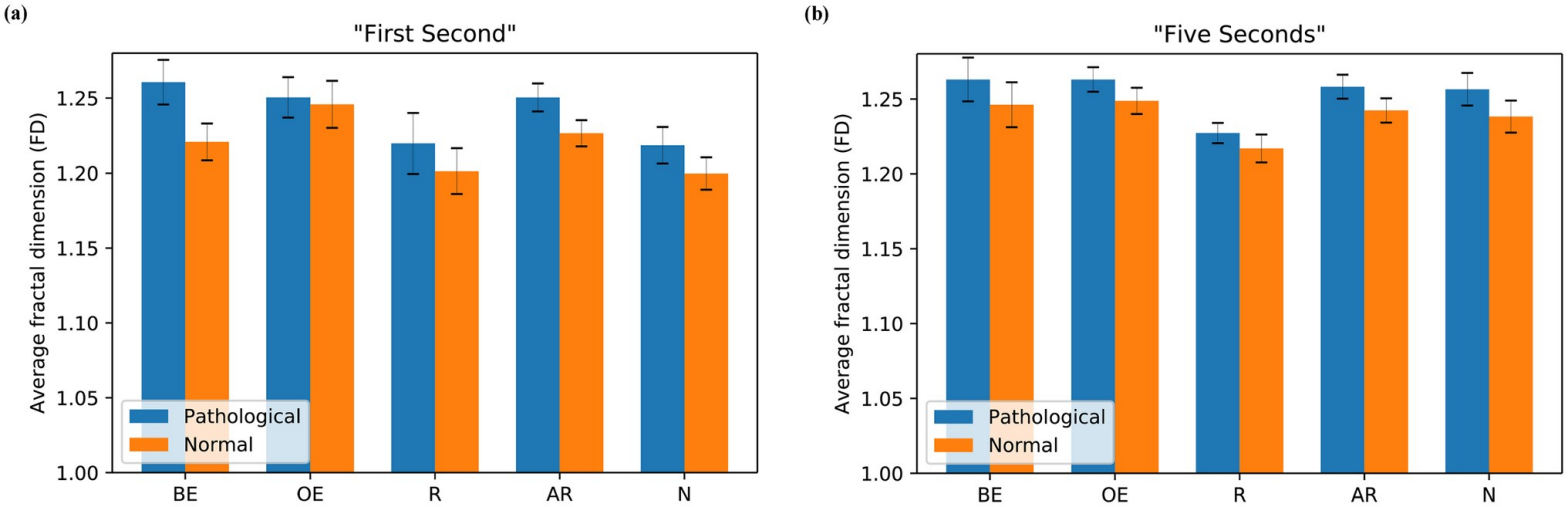
Average visual search complexity of scanpaths for pathological and normal stimuli across different groups of participants: Brain-experts (BE), Other-experts (OE), Registrars (R), All-radiologists (AR), and Naïves (N) (a) “First Second” and (b) “Five Seconds”.

Fig 6(a) displays the average FD of different groups of participants for pathological and normal stimuli of “First Second” analysis. For pathological stimuli, none of the statistical tests were significant. The relationship between Brain-experts and Registrars showed no significant variance across participants, (means (Ms) = 1.261, 1.220; standard errors (SEs) = 0.015, 0.020; respectively), *t*(18) = 1.432, *p* = 0.212, Cohen’s D effect size (*d*) = 0.74. Similar relationship was found between Brain-experts and Naïves, (Ms = 1.261, 1.218; SEs = 0.015, 0.012; respectively), *t*(39) = 2.144, *p* = 0.098, *d* = 0.70. Moreover, the similar relationship trend was observed between Other-experts and Registrars, (Ms = 1.250, 1.220; SEs = 0.014, 0.020; respectively), *t*(14) = 1.265, *p* = 0.227, *d* = 0.68; and between Other-experts and Naïves, (Ms = 1.250, 1.218; SEs = 0.014, 0.012; respectively), *t*(35) = 1.541, *p* = 0.212, *d* = 0.55. Finally, the measures between All-radiologists and Naïves were, (Ms = 1.250, 1.218; SEs = 0.009, 0.012; respectively), *t*(55) = 2.113, *p* = 0.098, *d* = 0.56.

Similarly, for normal stimuli, we see the same trend except for Other-experts which is comparatively higher than other groups. However, the averages are lower compared to averages for pathological stimuli. There was no significant difference between Brain-experts and Registrars, (Ms = 1.221, 1.201; SEs = 0.012, 0.015; respectively), *t*(18) = 0.834, *p* = 0.415, *d* = 0.43, and the relationship was similar between Brain-experts and Naïves, (Ms = 1.221, 1.200; SEs = 0.012, 0.011; respectively), *t*(39) = 1.240, *p* = 0.278, *d* = 0.40. Furthermore, the statistical measures between Other-experts and Registrars were similar fashion, (Ms = 1.246, 1.201; SEs = 0.016, 0.015; respectively), *t*(14) = 1.728, *p* = 0.177, *d* = 0.93; and between Other-experts and Naïves, (Ms = 1.246, 1.200; SEs = 0.016, 0.011; respectively), *t*(35) = 2.375, *p* = 0.116, *d* = 0.85. Finally, the values between All-radiologists and Naïves were, (Ms = 1.226, 1.200; SEs = 0.009, 0.011; *t*(55) = 1.953, *p* = 0.140, *d* = 0.52.

#### Analysis for “Five Seconds”

Fig 6(b) shows the average FD of different groups of participants for pathological and normal stimuli of “Five Seconds” analysis. For pathological stimuli, there was no significant difference between Brain-experts and Registrars groups, (Ms = 1.263, 1.227; SEs = 0.015, 0.007; respectively), *t*(18) = 1.380, *p* = 0.461, *d* = 0.71; and between Brain-experts and Naïves groups, (Ms = 1.263, 1.256; SEs = 0.015, 0.011; respectively), *t*(39) = 0.363, *p* = 0.897, *d* = 0.12. Also, the statistics were not significant between Other-experts and Registrars, (Ms = 1.266, 1.227; SEs = 0.008, 0.007; respectively), *t*(14) = 2.950, *p* = 0.053, *d* = 1.59; and between Other-experts and Naïves, (Ms = 1.266, 1.256; SEs = 0.008, 0.011; respectively), *t*(35) = 0.523, *p* = 0.897, *d* = 0.19. Finally, the values between All-radiologists and Naïves, (Ms = 1.258, 1.256; SEs = 0.008, 0.011; respectively), *t*(55) = 0.130, *p* = 0.897, *d* = 0.03.

The statistics for normal stimuli exhibited a similar trend like the pathological stimuli in “Five Seconds” analysis. However, the averages are lower than averages for pathological stimuli. There was no significant difference between Brain-experts and Registrars groups, (Ms = 1.246, 1.217; SEs = 0.015, 0.009; respectively), *t*(18) = 1.082, *p* = 0.734, *d* = 0.56; and between Brain-experts and Naïves participants, (Ms = 1.246, 1.238; SEs = 0.015, 0.011; respectively), *t*(39) = 0.438, *p* = 0.756, *d* = 0.14. In addition, there was no significant difference across participants between Other-experts and Registrars, (Ms = 1.249, 1.217; SEs = 0.009, 0.009; respectively), *t*(14) = 2.181, *p* = 0.234, *d* = 1.18; and between Other-experts and Naïves, (Ms = 1.249, 1.238; SEs = 0.009, 0.011; respectively), *t*(35) = 0.601, *p* = 0.756, *d* = 0.22. Last of all, the statistics between All-radiologists and Naïves were (Ms = 1.242, 1.238; SEs = 0.008, 0.011; respectively), *t*(55) = 0.312, *p* = 0.756, *d* = 0.08.

### Re-scaled range exponent

#### Analysis for “First Second”

**Fig 7:**
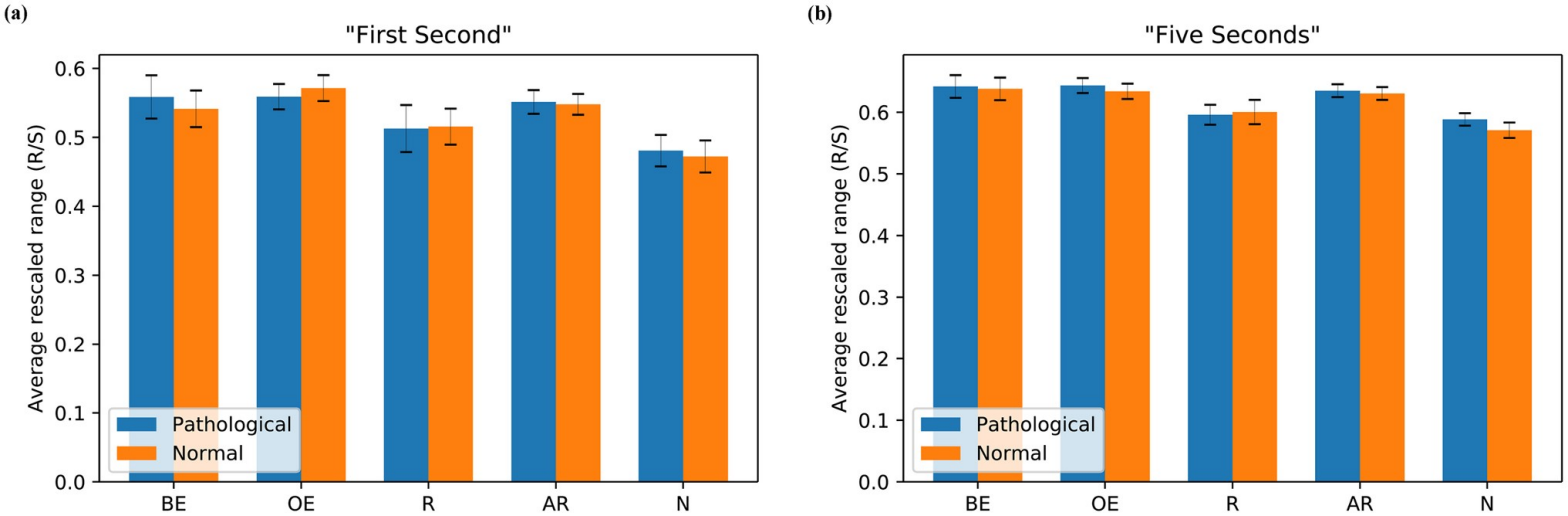
Average re-scaled range (R/S) exponent of scanpaths for pathological and normal stimuli across different groups of participants: Brain-experts (BE), Other-experts (OE), Registrars (R), All-radiologists (AR), and Naïves (N) (a) “First Second” and (b) “Five Seconds”.

Fig 7(a) exhibits the average R/S of different groups of participants for pathological and normal stimuli of “First Second” analysis. For pathological stimuli, the R/S between Brain-experts and Registrars participants was not significantly different, (Ms = 0.559, 0.513; SEs = 0.031, 0.034; respectively), *t*(18) = 0.785, *p* = 0.443, *d* = 0.41; and between Brain-experts and Naïves, (Ms = 0.559, 0.481; SEs = 0.031, 0.023; respectively), *t*(39) = 2.031, *p* = 0.082, *d* = 0.66. Also, it was not significant between Other-experts and Registrars, (Ms = 0.559, 0.513; SEs = 0.018, 0.034; respectively), *t*(14) = 1.303, *p* = 0.267, *d* = 0.70; and between Other-experts and Naïves, (Ms = 0.559, 0.481; SEs = 0.018, 0.023; respectively), *t*(35) = 2.100, *p* = 0.082, *d* = 0.76. Moreover, the R/S statistics between All-radiologists and Naïves were (Ms = 0.551, 0.481; SEs = 0.017, 0.023; respectively), *t*(55) = 2.512, *p* = 0.075, *d* = 0.67.

Similarly, the normal stimuli’s statistics were similar in pattern to the pathological stimuli. In this case, there was no significant difference between Brain-experts and Registrars, (Ms = 0.541, 0.516; SEs = 0.027, 0.026; respectively), *t*(18) = 0.529, *p* = 0.603, *d* = 0.27; and between Brain-experts and Naïves, (Ms = 0.541, 0.473; SEs = 0.027, 0.023; respectively), *t*(39) = 1.881, *p* = 0.113, *d* = 0.61. In addition, the R/S was not significantly different between Other-experts and Registrars, (Ms = 0.571, 0.516; SEs = 0.019, 0.026; respectively), *t*(14) = 1.691, *p* = 0.141, *d* = 0.91 but it was significantly different between Other-experts and Naïves, (Ms = 0.571, 0.473; SEs = 0.019, 0.023; respectively), *t*(35) = 2.610, *p* = 0.033; *d* = 0.94. Also, there was a significant difference between All-radiologists and Naïves, (Ms = 0.548, 0.473; SEs = 0.015, 0.023; respectively), *t*(55) = 2.804, *p* = 0.033, *d* = 0.75.

#### Analysis for “Five Seconds”

Fig 7(b) shows the average R/S of different groups of participants for pathological and normal stimuli of “Five Seconds” analysis. For the pathological stimuli, there was no significant difference between Brain-experts and Registrars, (Ms = 0.642, 0.596; SEs = 0.018, 0.016; respectively), *t*(18) = 1.367, *p* = 0.188, *d* = 0.71 but it was significantly different between Brain-experts and Naïves, (Ms = 0.642, 0.588; SEs = 0.018, 0.010; respectively), *t*(39) = 2.783, *p* = 0.014, *d* = 0.90. Furthermore, the R/S between Other-experts and Registrars was not significantly different, (Ms = 0.643, 0.596; SEs = 0.012, 0.016; respectively), *t*(14) = 2.242, *p* = 0.052; *d* = 1.21. However, we found a significant difference between Other-experts and Naïves in R/S, (Ms = 0.643, 0.588; SEs = 0.012, 0.010; respectively), *t*(35) = 3.152, *p* = 0.008, *d* = 1.13. Finally, the relationship between All-radiologists and Naïves was significantly different across participants, (Ms = 0.635, 0.588; SEs = 0.010, 0.010; respectively), *t*(55) = 3.175, *p* = 0.008, *d* = 0.84.

The trend of the normal stimuli statistics was similar to pathological stimuli. There was no significant difference in R/S between Brain-experts and Registrars groups, (Ms = 0.638, 0.600; SEs = 0.018, 0.020; respectively), *t*(18) = 1.105, *p* = 0.284, *d* = 0.57. However, there was a significant difference between Brain-experts and Naïves groups, (Ms = 0.638, 0.571; SEs = 0.018, 0.013; respectively), *t*(39) = 3.126, *p* = 0.008, *d* = 1.01. Likewise, no significant difference was found between Other-experts and Registrars, (Ms = 0.634, 0.600; SEs = 0.012, 0.020; respectively), *t*(14) = 1.479, *p* = 0.202, *d* = 0.80 but significant difference was observed between Other-experts and Naïves, (Ms = 0.634, 0.571; SEs = 0.012, 0.013; respectively), *t*(35) = 3.026, *p* = 0.008, *d* = 1.09. Also, it was significantly different in R/S between All-radiologists and Naïves, (Ms = 0.630, 0.571; SEs = 0.010, 0.013; respectively), *t*(55) = 3.708, *p* = 0.003, *d* = 0.99.

### Detrended fluctuation analysis exponent

#### Analysis for “First Second”

**Fig 8:**
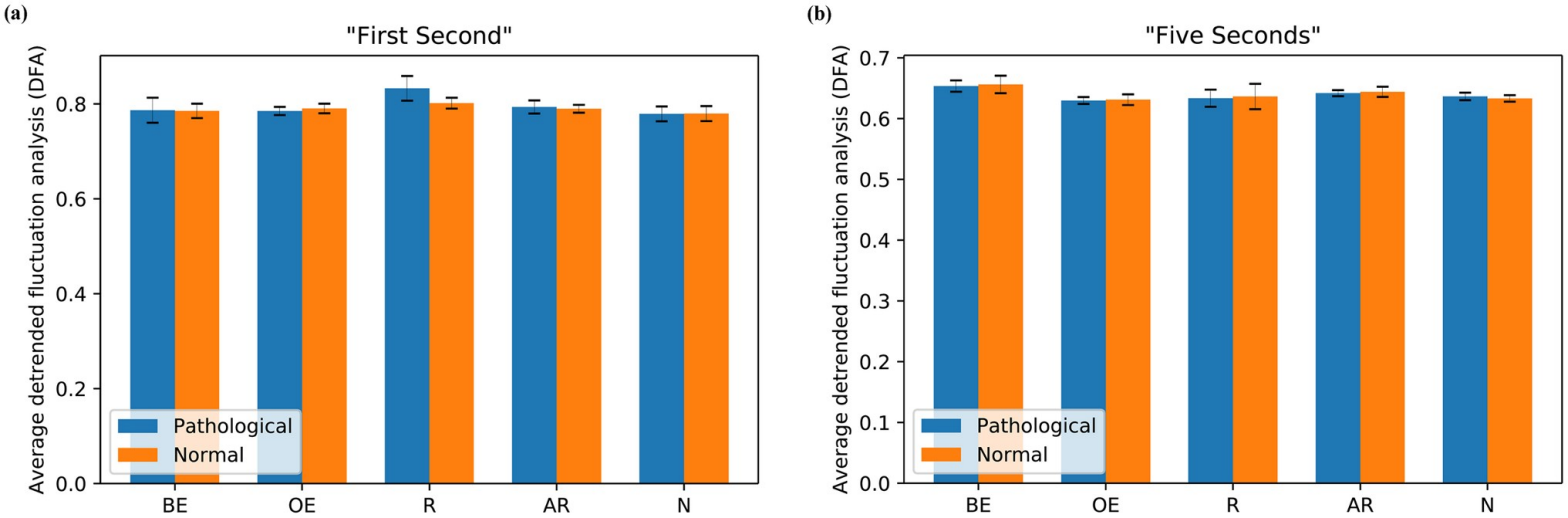
Average detrended fluctuation analysis (DFA) exponent of scanpaths for pathological and normal stimuli across different groups of participants: Brain-experts (BE), Other-experts (OE), Registrars (R), All-radiologists (AR), and Naïves (N) (a) “First Second” and (b) “Five Seconds”.

Fig 8(a) displays the average DFA of different groups of participants for pathological and normal stimuli of “First Second” analysis. For the pathological stimuli, we did not find any significant difference between Brain-experts and Registrars participants, (Ms = 0.787, 0.833; SEs = 0.026, 0.026; respectively, *t*(18) = 0.947, *p* = 0.806, *d* = 0.49; and between Brain-experts and Naïves, (Ms = 0.787, 0.779; SEs = 0.026, 0.016; respectively), *t*(39) = 0.267, *p* = 0.806, *d* = 0.09. Likewise, there was no significant difference between Other-experts and Registrars, (Ms = 0.785, 0.833; SEs = 0.009, 0.026; respectively), *t*(14) = 2.250, *p* = 0.206, *d* = 1.21; and Other-experts and Naïves, (Ms = 0.785, 0.779; SEs = 0.009, 0.016; respectively), *t*(35) = 0.247, *p* = 0.806, *d* = 0.09. Also, no significant difference was found between All-radiologists and Naïves, (Ms = 0.794, 0.779; SEs = 0.014, 0.016; respectively), *t*(55) = 0.701, *p* = 0.806, *d* = 0.19.

For the normal stimuli, there was no significant difference between Brain-experts and Registrars, (Ms = 0.785, 0.802; SEs = 0.015, 0.011; respectively), *t*(18) = 0.590, *p* = 0.813, *d* = 0.30; and between Brain-experts and Naïves, (Ms = 0.785, 0.780; SEs = 0.015, 0.016; respectively), *t*(39) = 0.238, *p* = 0.813, *d* = 0.08. Similarly, there was no significant difference between Other-experts and Registrars, (Ms = 0.790, 0.802; SEs = 0.010, 0.011; respectively), *t*(14) = 0.662, *p* = 0.8131; *d* = 0.36; and between Other-experts and Naïves, (Ms = 0.790, 0.780; SEs = 0.010, 0.016; respectively), *t*(35) = 0.425, *p* = 0.813, *d* = 0.15. Moreover, the DFA between All-radiologists and Naïves was not significantly different, (Ms = 0.790, 0.780; SEs = 0.008, 0.016; respectively), *t*(55) = 0.593, *p* = 0.813, *d* = 0.16.

#### Analysis for “Five Seconds”

Fig 8(b) shows the average DFA results of “Five Seconds” analysis for pathological and normal stimuli. For the pathological stimuli, no significant difference was discovered between Brain-experts and Registrars groups, (Ms = 0.653, 0.633; SEs = 0.009, 0.014; respectively), *t*(18) = 1.098, *p* = 0.656, *d* = 0.57; and between Brain-experts and Naïves groups, (Ms = 0.653, 0.636; SEs = 0.009, 0.006; respectively), *t*(39) = 1.576, *p* = 0.615, *d* = 0.51. Also, there was no significant difference between Other-experts and Registrars, (Ms = 0.630, 0.634; SEs = 0.006, 0.014; respectively), *t*(14) = 0.304, *p* = 0.765, *d* = 0.16; and between Other-experts and Naïves, (Ms = 0.630, 0.636; SEs = 0.006, 0.006; respectively), *t*(35) = 0.656, *p* = 0.656, *d* = 0.24. Moreover, the DFA was not significantly different between All-radiologists and Naïves, (Ms = 0.642, 0.636; SEs = 0.005, 0.006; respectively), *t*(55) = 0.640, *p* = 0.656, *d* = 0.17.

The normal stimuli bars shows that there was no significant difference between Brain-experts and Registrars, (Ms = 0.656, 0.636; SEs = 0.014, 0.021; respectively), *t*(18) = 0.712, *p* = 0.809, *d* = 0.37; and between Brain-experts and Naïves, (Ms = 0.656, 0.633; SEs = 0.014, 0.005; respectively), *t*(39) = 1.782, *p* = 0.413, *d* = 0.58. Likewise, no significant difference was found between Other-experts and Registrars, (Ms = 0.631, 0.636; SEs = 0.009, 0.021; respectively), *t*(14) = 0.281, *p* = 0.833, *d* = 0.15; and between Other-experts and Naïves, (Ms = 0.631, 0.633; SEs = 0.009, 0.005; respectively), *t*(35) = 0.212, *p* = 0.833, *d* = 0.08. Moreover, the DFA between All-radiologists and Naïves was not significantly different, (Ms = 0.644, 0.633; SEs = 0.008, 0.005; *t*(55) = 1.053, *p* = 0.743, *d* = 0.28.

## Discussion

### Necessity of simultaneous analysis

The FD in “First Second” pathological analysis suggests that the expert radiologists develop higher geometrical gaze complexity than Registrars and Naïves. However, the high geometrical gaze complexity of radiologists could be in a larger or smaller spatial coverage which cannot be confirmed until the “First Second” temporal through R/S and DFA analyses are done. However, the DFA analysis using “First Second” data is not correct due to difficulty of removing the large-scale trend with a small number of points. Thus, only the R/S analysis is valid for the “First Second” data which also illustrates that the experts have more focused gaze compared to the Registrars and Naïves. Similarly, the time series analysis through R/S can only provide a rough estimate regarding the focusing status of the scanpaths but cannot provide the movement complexity of scanpaths in detail. In particular, it can only reveal the focusing ability in terms of spatial regions but cannot reveal details about how the gaze moves around a spatial region. Therefore, the spatial and temporal analyses are simultaneously required to know the gaze behaviour more accurately. Thus, by considering both analyses of the FD and R/S simultaneously for the pathological stimuli, it can be suggested that the high geometrical complexity of an expert’s scanpath is within a smaller spatial coverage. As the analyses are for pathological stimuli, the experts may be focusing in the abnormal regions. These findings can be attributed to experts quickly focusing on abnormal regions, inspecting the regions with deep insights, with the aim to get detailed information regarding the abnormalities. On the other hand, the Registrars and Naïves could not focus quickly and inspect with deep insights to the abnormalities, they have random and high fluctuations gazes. In addition, the Registrars and Naïves also might miss abnormalities more than experts.

Other reasons Registrars achieve lower average FD scores may be their lack of sufficient training and their enthusiastic, learning mind which triggers them to find more false positive abnormalities resulting in a less focused gaze. However, could this phenomenon be captured by “time to initial hit” [36] parameter? The answer is “No” because the “time to initial hit” parameter can only estimate the percentage of being fixated by abnormalities during the initial viewing of radiologists. Although the “time to initial hit” provides valuable information about the initial perception of abnormality detection rate (about 57 percent of abnormalities having a 95 percent chance to be fixated in the first second of viewing), it does not allow one to differentiate the different patterns of visual behaviour among the different expertise levels of radiologists and cannot tell how an expert’s gaze moves around abnormalities after being fixated. Moreover, can another typical parameter “dwell time in ROI” [16, 23] measure the focusing ability of a scanpath? Although “dwell time in ROI” can provide important information about some particular objects/ROI (for example, both neurologists and controls spend similar time on large lesions), it cannot estimate the overall focusing characteristics of a scanpath in a score.

### Effect of stimuli in gaze patterns

The analyses for normal stimuli show a similar trend among the participants resembling pathological stimuli. However, the FDs are lower than pathological stimuli for all participants. The reason for the experts’ low FD is because the normal stimuli do not stimulate gaze as much as pathological stimuli due to the lack of abnormalities. As a result, the gaze does not create many small movements like the pathological stimuli. When a person’s gaze is attracted by an abnormal area, he/she becomes interested in it and examines it carefully in all parts resulting in many small movements and higher FD. The Registrars and Naïves visual search complexity for pathological stimuli are higher than normal stimuli, which may occur due to some clear abnormal regions attracting the gazes of those participants. On the contrary, the R/S exponents for normal stimuli are almost similar to pathological stimuli for all participants. The reason for this is because participants did not know which stimulus is normal or pathological, they performed a similar visual search strategy to find abnormalities and spent similar amount of time in some stimulus regions resulting in similar R/S exponents. This again provides evidence that both analyses are required to characterise visual search behaviour.

### Effect of viewing duration in gaze patterns

When analysing the full five seconds viewing duration, the results are similar to the “First Second” analyses. Specifically, when looking at pathological cases, participants exhibited similar behaviour between the “First Second” and “First Second” analyses, both spatially and temporally. However, Naïves participants exhibited higher values of visual search complexity. Naïves participants, on average, had higher FD and lower R/S and DFA values when considering the “First Second” analyses. This suggests that Naïves participants exhibited a larger search coverage as compared to experts and registrars. The reason why Naïves participants exhibited lower FD and R/S values when investigating the “First Second” analyses is because there were fewer saccades compared to “First Second” data, which cannot create a high FD value.

For normal stimuli, the FD values were higher in the “First Second” analyses for all participants. This suggests that participants exhibited a more exhaustive local search. For radiologists, this may be due to the possibility that they believe abnormalities may be present in the normal stimuli, which may lead to an increase in false-positive decisions. Because these stimuli do not capture radiologists’ attention in the first second (as detected by the FD result), viewing times should be limited to avoid the possibility for false-positive classification. Matsumoto *et al*. [23] noted that neurologists spent more time on clinically important areas with low saliency compared to controls. However, they did not explain the phenomenon of false positive results from viewing normal stimuli. In general, observation time increased (when comparing the results to the “First Second” to the “First Second” alone) for all the participants reflecting that their gaze was more focused.

### Overall summary

The expert radiologists exhibited different visual search characteristics than the Registrars and Naïves for both pathological and normal stimuli. The differences in some analyses were statistically significant specially for the R/S analyses. However, the differences in some analyses were not statistically significant but had large/medium effect sizes which confirm the meaningfulness of the analyses practically and clinically. The experts have a consistently focused high search complexity and the Naïves have a consistent, unfocused high search complexity. The difference in the normal stimuli suggests that the experts have different search behaviours from Registrars and Naïves regardless of whether or not an abnormality is present. Another important finding from “First Second” DFA analysis is that the Brain-experts were more focused than Other-experts. This provides evidence that the brain experts demonstrate a high level of expertise during viewing of brain stimuli and might have been achieved through intensive specialised training on brain stimuli. This also shows further that eye-tracking measures can be used as evidence for expertise, therefore, the quantification of the scanpath can offer valid surrogate biomarkers of higher cognitive function. As a result, it can be said that the expertise obviously guides the gaze of the experts. We considered the “First Second” results for this finding because these can be considered as a steady state response of the participants whereas the “First Second” results are a transient response. We also neglected FD analyses for this finding as other experts are also experts in radiology and they should have similar visual search complexity. Moreover, the HE analyses suggest that experts had memory for image pathology and structure which was achieved through highly specialised training.

### Limitations

Despite the rigorous analyses of visual search patterns through three parameters, the study has some limitations. We presented 2D brain stimuli, rather than a volumetric stack of images at different scan planes of the brain, which is not a realistic clinically viewing scenario. In addition, the DFA algorithm cannot give a correct estimate of the fluctuation function with a smaller number of points as the linear de-trending performs poorly in this scenario [35]. Therefore, the analysis for the “First Second” contains some artefacts. Further research will be aimed to analyse real clinical viewing scenarios and extend our findings to other types of stimuli (e.g., mammograms and non-medical images).

## Conclusion

In this study, we investigated the visual search characteristics of groups of radiologists with different experience levels and Naïves in both the spatial and time domains for brain stimuli. An analysis of both the spatial and time domain are simultaneously required to accurately understand characteristics of how radiologists observe stimuli, providing important information about where and how radiologists see medical images. This hypothesis has been validated through our extensive experiments by using appropriate parameters FD, R/S and DFA. This improves upon prior work where the analysis was done in a single domain and to our knowledge, is the first research effort to examine brain stimuli using these methods. These experts’ characteristics manifest as greater focus than Registrars and Naïves. Moreover, experts inspect abnormalities in greater detail to determine specific types of them. Brain-experts show higher expertise than Other-experts in brain stimuli due to their specific domain knowledge. A normal stimulus may progressively persuade radiologists to focus on false positive results at times, thus, longer exposures may exacerbate such biases. The “First Second” and “First Second” analyses show that experts have similar behaviour over time whereas Naïves have different behaviour.

Trainees can learn the visual search techniques of experts from our study and apply those techniques to improve their expertise quickly. In future work, we will try to generate filtered heat maps using sliding FD, R/S and DFA parameters to train a machine learning model to predict scanpaths and regions of interest in images.
